# Time to death and its predictors among HIV patients on antiretroviral therapy in public health facilities of Horro Guduru Wallaga zone, Ethiopia: a retrospective cohort study

**DOI:** 10.3389/fpubh.2025.1565573

**Published:** 2025-04-16

**Authors:** Benti Morka, Firezer Belay Keno, Dejene Seyoum Gebre, Worku Fikadu, Gemechu Tiruneh, Eba Abdisa Golja, Adisu Ewunetu

**Affiliations:** ^1^Horro Guduru Wallaga Zone Health Department, Shambu, Ethiopia; ^2^School of Public Health, Institute of Health Science, Wallaga University, Nekemte, Ethiopia; ^3^School of Nursing and Midwifery, Institute of Health Sciences, Wallaga University, Nekemte, Ethiopia

**Keywords:** time to death, predictors, antiretroviral therapy, Horro Guduru Wallaga, Ethiopia

## Abstract

**Background:**

People with human immunodeficiency virus (HIV)/acquired immunodeficiency syndrome (AIDS) continue to die at substantial rates, even in our nation of Ethiopia, despite receiving antiretroviral medication. Limited evidence is available regarding these individuals’ time to death and its predictors. Therefore, this study aimed to evaluate time to death and its predictors among HIV/AIDS patients receiving antiretroviral therapy (ART) in this study area.

**Objective:**

The objective of the study was to assess time to death and its predictors among HIV patients receiving ART in public facilities of the Horro Guduru Wallaga (HGW) zone, Western Ethiopia, 2024.

**Methods and materials:**

A facility-based retrospective study was conducted involving 538 HIV-positive patients on anti-retroviral therapy. A simple random sampling method was used to select a sample from patient registrations between October 2018 and October 2023. Data were entered into EpiData version 3.1 and exported to STATA version 14. The Kaplan–Meier curve was used to estimate the survival probability after ART initiation. The Cox regression model was used to identify independent predictors of death. Significantly associated variables were reported with a *p*-value of less than 0.05 and the adjusted hazard ratio (AHR) with a 95% confidence interval.

**Results:**

Among the 538 study participants included in the final analysis, 42 (8%) individuals died. The finding of this study revealed that the incidence rate of HIV-related death was 2.81 deaths per 1,000 person-months. Diarrhea (AHR = 4.54; 95% CI 1.85–11.13), failure to take TB prophylaxis (AHR = 5.61; 95%CI: 2.25, 14.03), non-utilization of condoms (AHR = 2.62; 95% CI: 1.13, 6.08), and WHO clinical stages III and IV (AHR = 7.02; 95%CI: 3.11, 11.84) were identified as predictors of death among the patients.

**Conclusion:**

The time to death among HIV patients on ART in this study area was higher compared to the national HIV-related death report. A history of diarrhea, failure to adhere to tuberculosis prophylaxis, non-utilization of condoms, and HIV clinical stages III and IV were found to be predictors of time to death related to HIV. Therefore, it is important to promote behavioral changes, such as condom utilization, adherence to TB prophylaxis, and effective treatment of comorbid infections, to improve the lifespan of HIV patients.

## Introduction

Acquired immunodeficiency syndrome (AIDS) is an infectious disease caused by the human immunodeficiency virus (HIV) that predominantly affects an individual’s immune system (1). Globally, there have been 39.0 million people living with HIV (PLHIV), 40.4 million deaths since the start of the epidemic, and 1.3 million new infections. Additionally, only 29.8 million people with access to medicine have been reported. Approximately 20.8 million PLHIV cases have been reported in Africa, with 500,000 new infections and 260,000 deaths occurring annually (2). Not all patients respond optimally to antiretroviral therapy (ART), leaving them at risk of morbidity and mortality (3). Globally, AIDS-related illnesses claimed the lives of approximately 630,000 people in 2022, which is a decrease from 2.0 million people in 2004 and 1.3 million people in 2010. Since 2010, there has been a 55% decrease in AIDS-related mortality among women and girls and a 47% decrease among men and boys (2).

Approximately 23.8 million individuals are living with HIV across Africa. Each year, over a million adults and children lose their lives to HIV/AIDS in the continent (4). Tanzania, Uganda, and Ethiopia have already surpassed the 2020 goal of reducing mortality by 75%. Ethiopia, Rwanda, and Uganda have incidence-prevalence ratios of less than 0.03, indicating that these nations are on track to eliminate HIV by 2030. Due to its high mortality rate, Ethiopia has an incidence-mortality ratio of less than 1. While the country is on target to achieve the 2020 national objective, it will fall short of the goal for individuals aged 15–49 years (5). In Ethiopia, there are 11,000 deaths each year, with the majority (67%) occurring in individuals under 30 years (6). Although the nation has made significant progress in lowering the death rate by reducing its prevalence and incidence rates, it has proven difficult to monitor deaths related to HIV (7).

Previous research has found that patients on ART in resource-constrained areas face a significant risk of early mortality due to various factors, including sociodemographic characteristics such as age (8–11), educational status (8, 10), and residence (1, 12), all of which are directly associated with mortality in HIV/AIDS patients on ART. In addition, clinical and laboratory-related factors, including adherence to TB prophylaxis (1, 13), CD4 count (9, 11, 14), hemoglobin level (15–17), adherence status (1, 11, 13), WHO clinical stages (8, 9, 12, 18), functional status (16, 18, 19), and opportunistic infections (11, 14, 20), were associated with mortality in adult HIV/AIDS patients on ART. Moreover, behavioral and lifestyle factors, including, substance use (21, 22), were directly associated with time to death among HIV/AIDS patients on ART.

The majority of studies conducted in Ethiopia on this issue have been limited to a single hospital. However, this study aimed to include experiences from different public health facilities providing HIV services. Furthermore, there are no significant data regarding the incidence of the death rate and its predictors in this study setting. As a result, the purpose of this study was to investigate the incidence of time to death and its predictors among HIV patients on ART in selected public health facilities in the Horro Guduru Wallaga (HGW) zone. The findings of this study will help program managers and health planners in designing better strategies and making informed policy decisions and serve as a source of knowledge and insight to provide appropriate standard operating guidelines. Furthermore, the findings will help draw significant recommendations and provide baseline information and references for other researchers interested in this study area.

## Methods and materials

### Study area

The study was conducted in the HGW zone, in selected public ART clinics in Oromia, western Ethiopia. The administrative town of the zone, Shambu, is located 314 km west of Addis Ababa, at a latitude of 9°10′N to 9°50′N and a longitude of 36°00′E to 36°50′E. It covers a total land area of 8,097 km^2^ (23), and the zone has a total population of 862,369 (male: 431184 (49.9%); female: 431185 (50.1%)).

Approximately 89% of the population lives in rural areas, with most deriving their livelihoods from agriculture. The zone has 13 districts, 12 rural and 1 urban. A total of 15 public ART health clinics provide care for patients with chronic HIV. For this study, five ART facilities were randomly selected to examine time to death and its predictors. As of the June 2014 fiscal year report, there were 2,894 HIV patients in the zone. Among these patients, 114 were below 14 years of age and 2,780 were above 15 years of age (HGW Zone Health Department Report). This study was designed to evaluate time to death and its predictors during the study period.

### Study design and period

A facility-based retrospective cohort study design was employed using patient registration cards from 15 January 2024 to 15 February 2024.

### Population

All patients with HIV infection on ART at the public facilities in the HGW zone were the source population, and HIV-infected patients who started antiretroviral therapy (ART) from October 2018 to October 2023 at these public facilities were the study population.

### Eligibility criteria

All HIV-infected patients aged 15 years and above, who were on antiretroviral therapy (ART) at the selected public hospital in the HGW zone from October 2018 to October 2023, were included. Patients with incomplete data on outcome variables, such as the date of treatment initiation, age of the patients (for those under 15 years), and other pertinent variables, were excluded from the study.

### Sample size determination

Using the Freedman formula method for sample size determination, the sample was calculated as follows:


$$E=\frac{{\left(Z\frac{\alpha }{2}+Z\beta \right)}^{2}}{{\left(\mathrm{lnHR}\right)}^{2}P\left(1-P\right)}$$

Based on a previously conducted study, three variables—functional status, adherence to TB prophylaxis, and hemoglobin level—were identified as predictors of mortality among HIV patients on ART. The sample size required to achieve 80% power (*β* = 0.20) at a 5% (*α* = 0.05) significance level, after assuming that the highest sample size was taken for the functional status predictor with an estimate of 538 participants, was calculated using the Freedman formula (24). The detailed sample size calculations are presented in Table 1.

**Table 1:** Sample size calculation to estimate time to death and its predictors of HIV patients in public facilities of Horro Guduru Wallaga zone, Western Ethiopia, 2024.

Significant predictors	Z$\frac{\alpha }{2}$	Zβ	HR	E	Pr(E)	Sample size	Plus 10% non-response rate	Reference
Functional status	1.96	0.84	7.4	29	0.0593	489	538	(24)
TB prophylaxis	1.96	0.84	3.98	249	0.66	377	415	(1)
Hemoglobin level	1.96	0.84	2.76	69	0.182	379	417	(25)

### Sampling procedures and techniques

**Figure 1:**
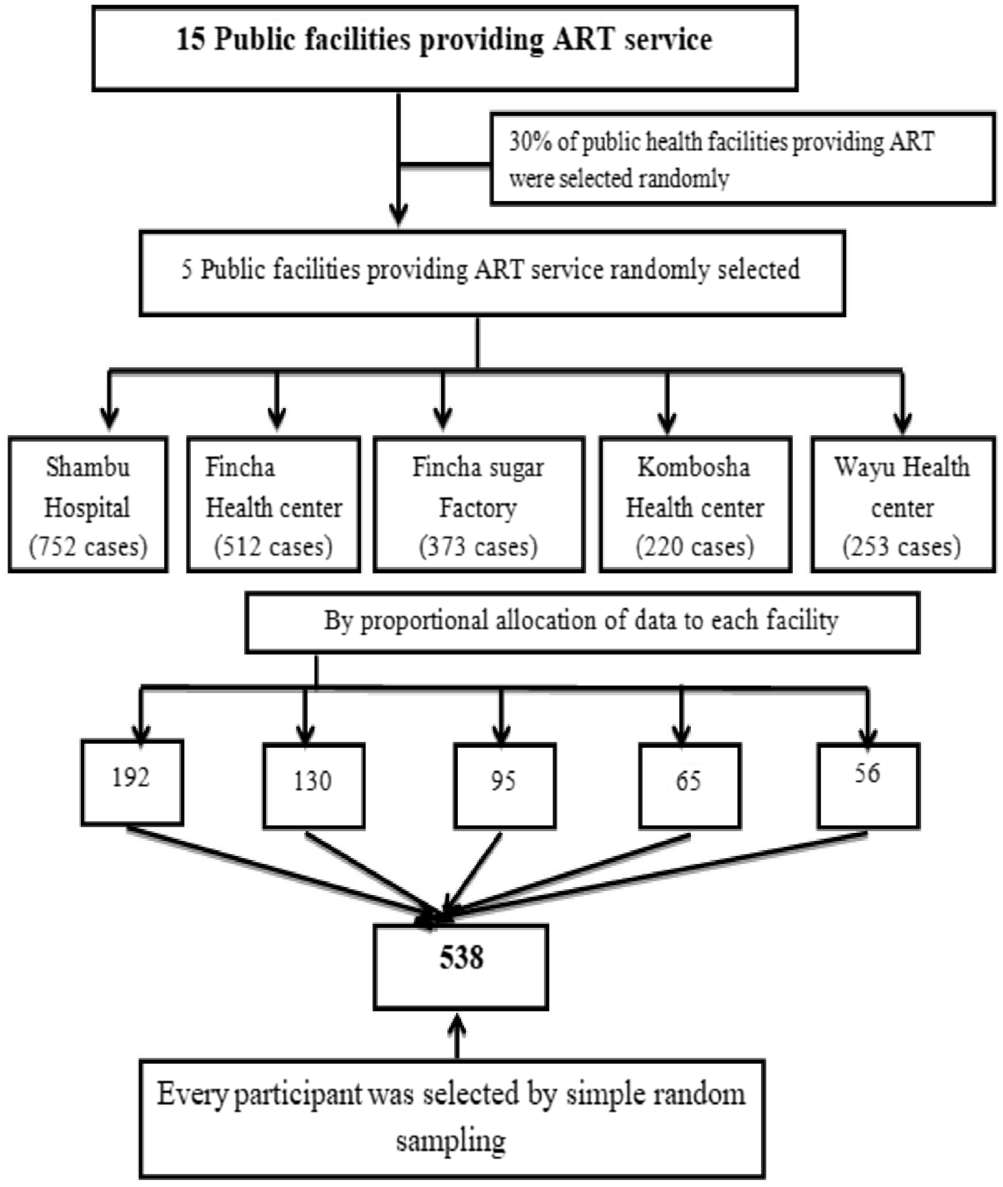
Sampling technique for the study on time to death and its predictors among the HIV patients on ART at the public facilities of the Horro Guduru Wallagazone, Western Ethiopia, 2024.

A simple random sampling method was used to select study participants from current HIV patients on ART at the selected public facilities in the zone. According to the annual HIV clinical reports, there are approximately 2,110 patients receiving care at the selected public ART facilities in the zone, which served as the sampling frame for this study. The distribution of patients across facilities was as follows: 752 from Shambu General Hospital, 512 from Fincha Health Centre, 373 from Fincha Sugar Factory Health Centre, 253 from Wayu Health Center, and 220 from Kombosha Health Centre. These patients that are currently on treatment formed the sampling frame for the study. A total of 538 study participants were selected from the ART clinics in the zone. Based on the proportionally allocated sample size for each facility, participants were randomly selected using the lottery method from the sampling frame, as shown in Figure 1.

### Variables of the study

#### Dependent variable

- The dependent variable was time to death.

#### Independent variables

Sociodemographic factors: sex, age, marital status, educational status, occupation, residence, and family size.

Clinical and laboratory-related factors: TB prophylaxis, CD4 count, VL status, hemoglobin level, functional status, ART regimen, adherence status, cotrimoxazole preventive therapy, WHO clinical stage, functional status, BMI status, side effects, HIV advanced stage, and opportunistic infections.

Behavioral and lifestyle-related factors: Alcohol consumption, smoking, condom utilization, khat chewing, and substance abuse.

### Operational definition

Time to death: The time at which an HIV patient died during the follow-up period after initiating antiretroviral treatment. (1)

#### Adherence

Good adherence: 95% or greater adherence, defined as missing no more than 1 dose out of 30 or no more of 2 doses out of 60.

Fair adherence: 85–94% adherence, defined as missing 2–4 doses out of 30 or missing 4–9 doses out of 60.

Poor adherence: Less than 85% adherence, defined as missing 5 doses out of 30 or more than 10 doses out of 60 (25).

Incidence of death: The number of deaths among new cases, divided by the total person-time at risk in months, and expressed per 1,000 person-months (13).

Survival time: In this study, survival time was defined as the length of time between ART initiation and either death or censoring.

Censored: At the conclusion of the study, the patients were considered censored if they had moved to different medical facilities, were lost to follow-up, or were still alive.

Event: The death of the HIV patients after the initiation of ART (1, 11).

### Data collection tools

Data collection tools were adapted from different studies (22, 24, 26). The checklist was initially prepared in English based on the standardized ART registers, the intake form, and follow-up format. It was then translated into Afaan ‘Oromo’ and subsequently backtranslated into English. It contained sociodemographic information, behavioral and lifestyle factors, and clinical and laboratory data. The starting point for the retrospective follow-up was the initiation of ART, while the endpoint was the date of death, the date of lost to follow-up, the date of transfer to another facility, or the date of last contact, up until 30th October 2023. Before collecting the data, the medical records were reviewed (both baseline and follow-up records). Death was confirmed using the ART registration follow-up cards by identifying the patient’s medical record number. The most recent laboratory test results prior to the initiation of ART were used as baseline values.

### Data processing and analysis

After data cleansing and completeness checks, the data were entered into EpiData version 3.02. Cross-tabulation was used to identify any inconsistencies. Data were then transferred to Stata 14.0 for analysis. The main outcome of this study was death or censorship due to all AIDS-related causes. The outcome for each participant was dichotomized as either censoring or death. The time to mortality and its predictors were used to assess the association between the patients’ characteristics and the time from ART initiation to death. A univariate analysis was used to describe the patients’ baseline characteristics. A life table was used to estimate the time to death and its predictors after the initiation of ART.

The Kaplan–Meier curve and log-rank test were used to compare survival curves. Cox proportional hazards regression was used to determine independent predictors of time to death and significant variables associated with time to death. A bivariable Cox proportional hazards model was used to identify candidate variables for multivariable Cox regression. Variables with a *p*-value of less than 0.25 in the bivariable analysis were selected as candidates for the multivariable Cox regression analysis. A multivariable Cox proportional hazards model was used to identify predictors of time to death in the HIV-infected patients. The adjusted hazard ratio (AHR) with a 95% confidence interval (CI) and a *p*-value of less than 0.05 were used to determine the strength of association and statistical significance. Finally, the data were presented using tables, figures, and graphs.

### Data quality assurance and control

To ensure the quality of the data, the principal investigator conducted a one-day training session for the data collectors and supervisors on the basic principles of research ethics and the overall technique of data collection, ensuring adherence to the protocol. A pre-test was performed on 5% of the sample at the Bako Health Centre before the actual data collection to examine the accuracy of responses and estimate the time required to complete one questionnaire. Data quality checks were performed using EpiData version 3.02 for data entry, data exploration, simple frequency, consistency checks, and sorting techniques. Appropriate statistical models were used, and an assumption was verified for each model. A comparable data source was used for the discussion and conclusion.

## Results of the study

### Sociodemographic characteristics of the participants

A total of 538 HIV-infected individuals’s registration cards were included in the present study, with more than half of the participants (324, 59.85%) being female. The mean age of the patients was 33.74 years, with an SD of ±11.13 years. Approximately one-fourth of the participants were younger than 27 years old. The majority of the patients, 241 (44.8%), had no formal education, while 191 (35.5%) had primary education. Regarding occupational status, more than half of them were farmers, followed by daily laborers (134, 24.91%). The majority of the participants, 324 (60.22%), were from rural areas, and 214 (39.78%) of the participants were living with their parents. (Table 2).

**Table 2:** Socio-demographic characteristics of HIV patients on ART at public health facilities of Horro Guduru Wallaga, Western Ethiopia, 2024.

Variable		Frequency	Percent (%)
Age category	<27 years	138	25.65
	28–35 years	185	34.39
	36-42 years	112	20.82
	>43 years	103	19.14
Sex	Male	216	40.15
	Female	322	59.85
Education level	No education	241	44.80
	Primary school	191	35.50
	Secondary school	66	12.27
	Tertiary	40	7.43
Occupation status	Farmer	307	57.07
	Merchant	58	10.78
	Government employee	31	5.76
	Non-Governmental employee	8	1.49
	Daily laborer	134	24.91
Marital status	Never married	68	12.64
	Married	409	76.02
	Separated	10	1.86
	Divorced	51	9.48
Family size	1–3	406	75.46
	4–6	90	16.73
	≥7	42	7.81
Residence	Urban	214	39.78
	Rural	324	60.22

### Clinical and laboratory-related factors

Regarding clinical and laboratory characteristics, 19 (3.53%) of the reviewed patients had candidiasis, 30 (5.53%) had HIV-related diarrhea, three (4.23%) had Pneumocystis pneumonia (PCP), and 70 (13.01%) had wasting syndrome. Approximately two-thirds of the patients, 346 (64.31%), had a viral load of less than 1,000 copies/ml, while 23 (4.28%) had a viral load greater than 1,000 copies/ml, and 196 (31.41%) were not evaluated. Furthermore, 65 (12.08%) participants had a BMI < 16 kg/m^2^, approximately one-fifth had a BMI of 16–18.5 kg/m^2^, and more than two-thirds (362, 67.29%) had a BMI > 18.5 kg/m^2^.

Regarding WHO staging status, approximately 472 (87.73%) participants were in WHO stages I and II, whereas 66 (12.27%) participants were in WHO stages III and IV. Similarly, approximately two-thirds of the patients, that is, 506 (94.05%), had a working functional status, and 27 (5.05%) were ambulatory. More than two-thirds of the patients, that is, 342 (69.33%), were on tuberculosis preventive therapy, while 165 (30.67%) were not on TB prophylaxis. Furthermore, one-fourth of the individuals, that is, 112 (20.82%), had a history of opportunistic illness, while 308 (57.25%) had received CPT prophylaxis. In terms of functional state, approximately 489 (90.89%) participants were working, whereas 44 (8.17%) and 5 (0.92%) participants were bedridden and ambulatory, respectively (Table 3).

**Table 3:** Clinical and laboratory-related predictors of death among HIV patients on ART in public facilities of Horro Guduru Wallaga zone, Western Ethiopia, 2024 (*n* = 538).

Variable	Category	Frequency	Percent (%)
Candidiasis	Yes	19	3.53
	No	519	96.47
Cryptococci meningitis	Yes	1	0.19
	No	537	99.81
Kaposi Sarcoma	Yes	1	0.19
	No	537	99.81
HIV related diarrhea	Yes	30	5.58
	No	508	94.42
Encephalopathy	Yes	3	0.56
	No	535	99.44
Herpes simplex	Yes	4	0.74
	No	534	99.26
Herpes zoster	Yes	4	0.74
	No	534	99.26
Mycosis	Yes	5	0.93
	No	533	99.07
Pneumocystis pneumonia (PCP)	Yes	23	4.28
	No	517	95.72
Wasting Syndrome	Yes	70	13.01
	No	468	86.99
Viral load detection	≤1,000	346	64.31
	>1,000	23	4.28
	Not done	196	31.41
TB prophylaxis	Yes	373	69.33
	No	165	30.67
CPT prophylaxis	Yes	377	70.07
	No	161	29.93
BMI status	<16 kg/m^2^	65	12.08
	16–18.49 kg/m^2^	111	20.63
	≥18.5 kg/m^2^	362	67.29
Drug side effect	Yes	28	5.20
	No	510	94.80
Regimen	1^st^ line	529	98.33
	2^nd^ regimen	9	1.67
WHO staging	Stages 1 & 2	472	87.73
	Stage 3 &4	66	12.27
Hemoglobin level	≤10gm/dl	26	4.83
	>10gm/dl	168	31.23
	Not done	344	63.94

**Figure 2:**
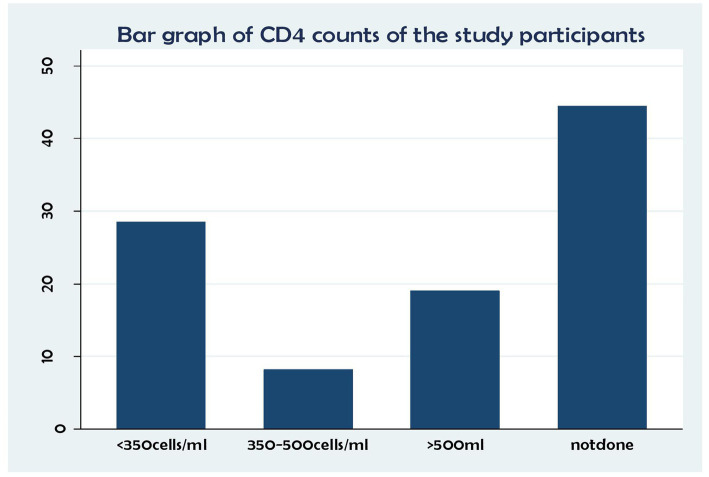
Association between CD4 status and time to death and its predictors among the HIV patients on ART at the public health facilities in the HGW Zone, Western Ethiopia, 2024 (*N* = 538).

In terms of CD4 status, of the patients with fewer than 350 cells/ml copies, 153 (28.44%) and 44 (8.18%) had between 350 and 500 cells/copies, 102 (18.98%) had no results, and 239(44.42%) had more than 500 copies/ml (Figure 2).

**Figure 3:**
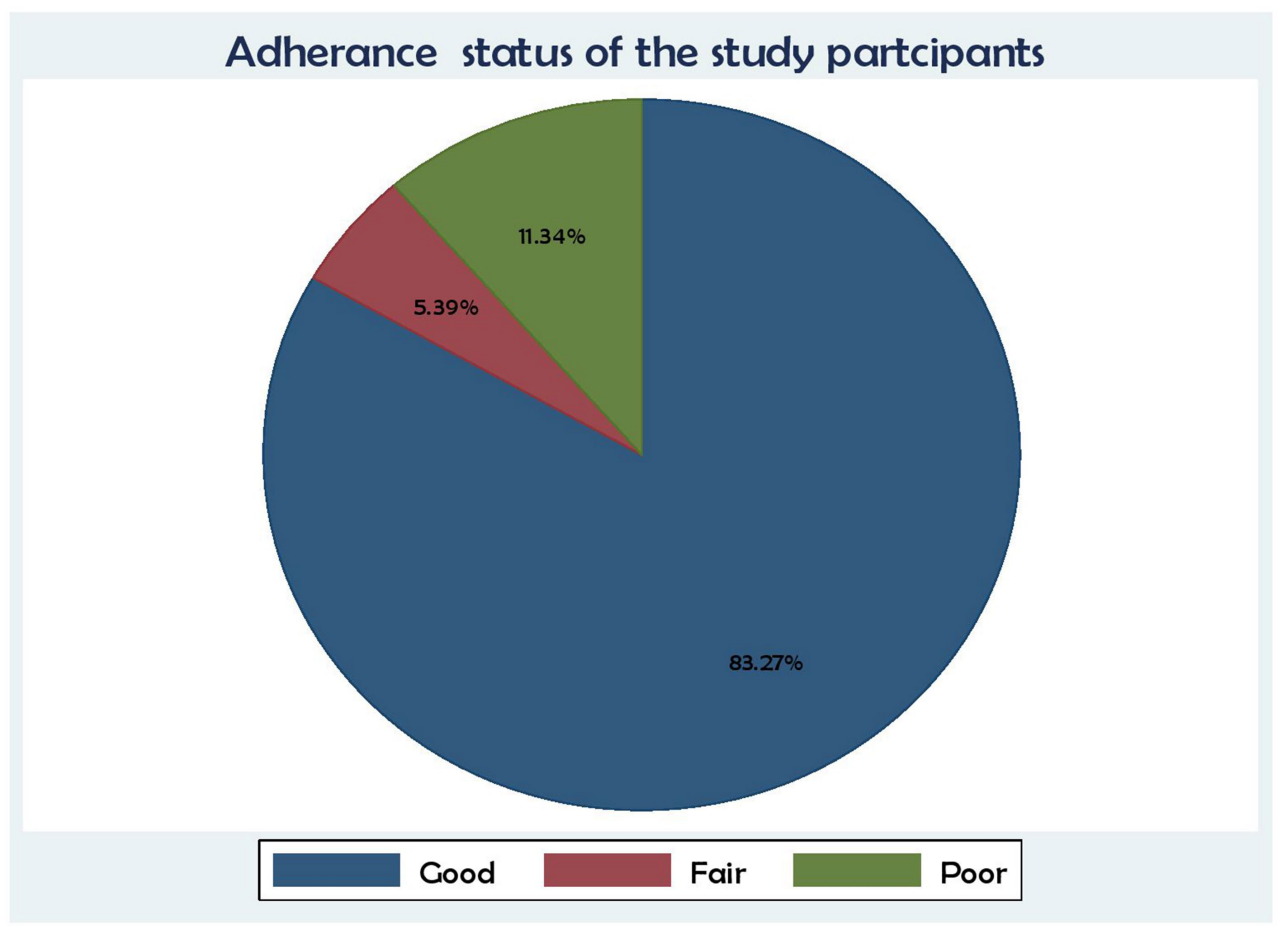
Association between ART adherence status and time to death and its predictors among the HIV patients on ART at the public health facilities in the HGW Zone, Western Ethiopia, 2024 (*N* = 538).

In terms of the study participants’ adherence level, 448 (83.27%) had good adherence, 61 (11.34%) had poor adherence, and 29 (5.39%) had fair adherence to their medication (Figure 3).

**Figure 4:**
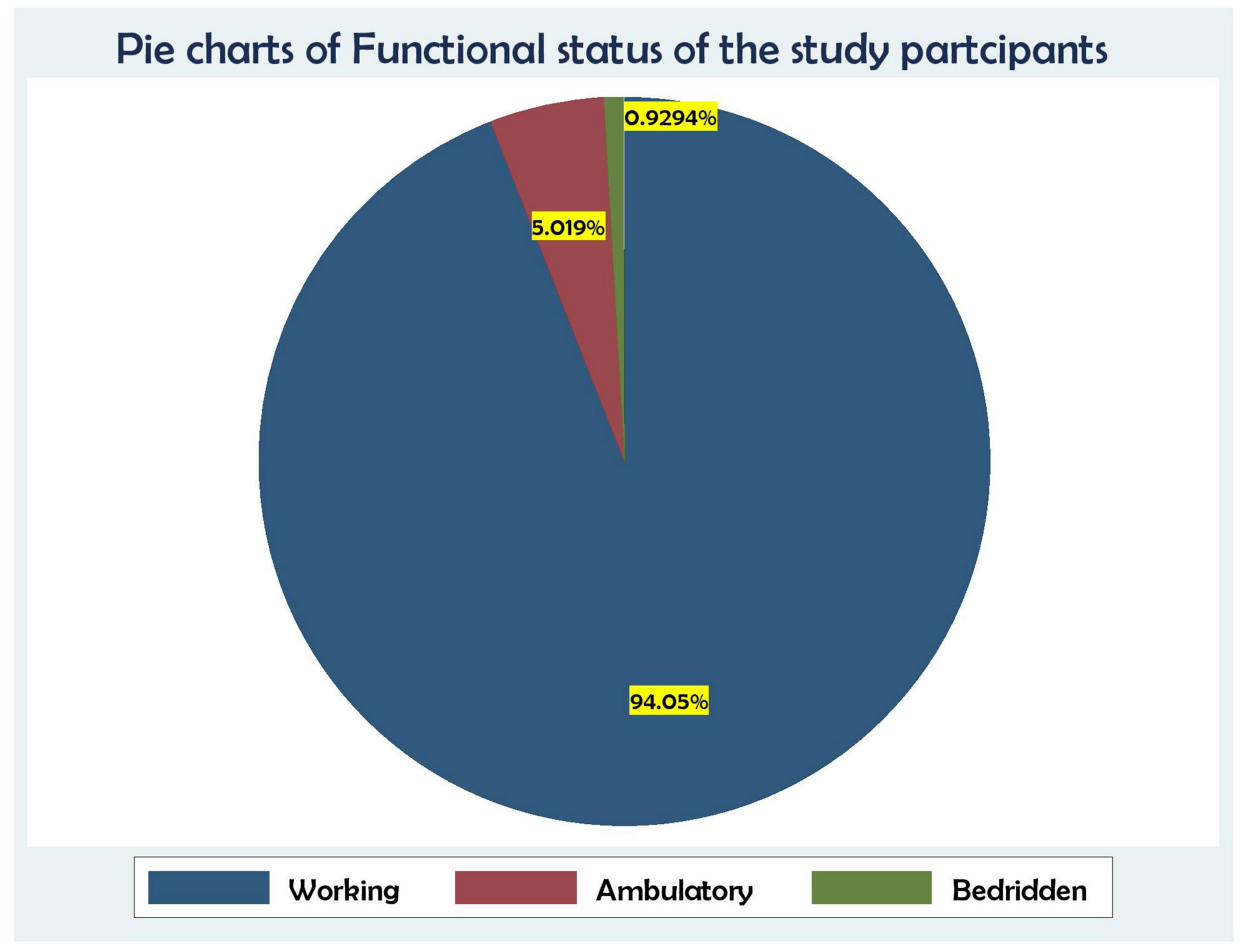
Association between functional status and time to death and its predictors among the HIV patients on ART at the public health facilities in the HGW Zone, Western Ethiopia, 2024 (*N* = 538).

Regarding functional status, 506 (94.05%) were working, followed by 27 (5.02%) who were ambulatory and 5 (0.93%) who were bedridden (Figure 4).

### Behavioral and lifestyle-related factors

A total of 322 participants (89.85%) had used condoms in the past. Only 22 (4.09%) of the individuals had a history of cigarette smoking, while 55 (10.23%) had a history of alcohol consumption. Approximately 31 (5.76%) participants had a history of substance use (Table 4).

**Table 4:** Behavioral and life style related characteristics of patients on ART in public facilities of Horro Guduru Wallaga zone, Western Ethiopia, 2024 (*n* = 538).

Variable	Category	Frequency	Percent (%)
Condom utilization	Yes	322	59.85
	No	216	40.15
Smoking cigarettes	Yes	22	4.09
	No	516	95.91
Substance use	Yes	31	5.76
	No	507	91.24
Disclosure status	Yes	492	91.45
	No	46	8.55
Alcohol intake	Yes	55	10.22
	No	483	89.88

### Survival characteristics after the initiation of ART

After the initiation of ART, the HIV-infected patients were followed for a period ranging from 6 months to 60 months, which provided a total of 14,907 person-months of observation. At the end of the follow-up period, 466 (86.62%) were alive, 11 (2.04%) were lost to follow-up, 19 (3.53%) were dropped, and 42 (7.81%) were reported dead due to HIV/AIDS. Of a of total 42 deaths, 10 (23.80%) occurred within the first 6 months of the follow-up and 18 (42.85%) occurred within the first 12 months of ART initiation. The incidence rate of death was 2.81 deaths per 1,000 patient-months (95%CI: 2.08; 3.81) during the follow-up period (Table 5).

**Table 5:** Life table/Cumulative proportional surviving at the end of Interval of patients on ART in public facilities of Horro Guduru Wallaga zone, Western Ethiopia, 2024.

Interval	Beginning Total	Deaths	Lost	Survival	Standard Error	[95% Conf. Int.]	
0–6	538	10	68	0.9802	0.0062	0.9634	0.9893
6–12	460	4	50	0.9711	0.0076	0.9517	0.9828
12–18	406	4	61	0.9608	0.0091	0.9383	0.9752
18–24	341	3	44	0.9518	0.0104	0.9265	0.9685
24–30	294	2	53	0.9446	0.0115	0.9171	0.9632
30–36	239	6	36	0.9190	0.0152	0.8833	0.9441
36–42	197	6	34	0.8884	0.0192	0.8443	0.9205
42–48	157	4	44	0.8620	0.0227	0.8105	0.9004
48–54	109	1	52	0.8517	0.0247	0.7956	0.8934
54–60	56	1	38	0.8286	0.0330	0.7521	0.8834
60–66	17	1	16	0.7366	0.0916	0.5072	0.8713

### Time to death and comparisons

Time to death and comparisons of survival status between the categories of predictors were analyzed using the Kaplan–Meier curve and log-rank test. Of the 538 total study participants under ART follow-up, approximately 42 (8%) participants experienced an event (i.e., death).

**Figure 5:**
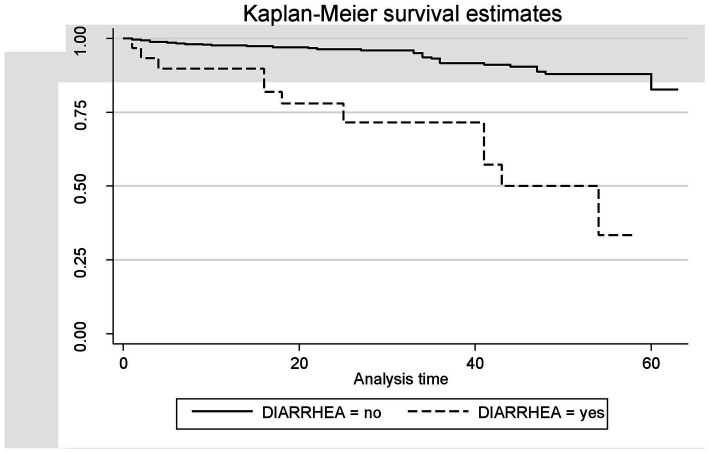
The Kaplan–Meier survival estimates based on the presence of HIV-related diarrhea in the retrospective study of the HIV patients on ART at the public facilities in HGW, Western Ethiopia, 2024.

**Figure 6:**
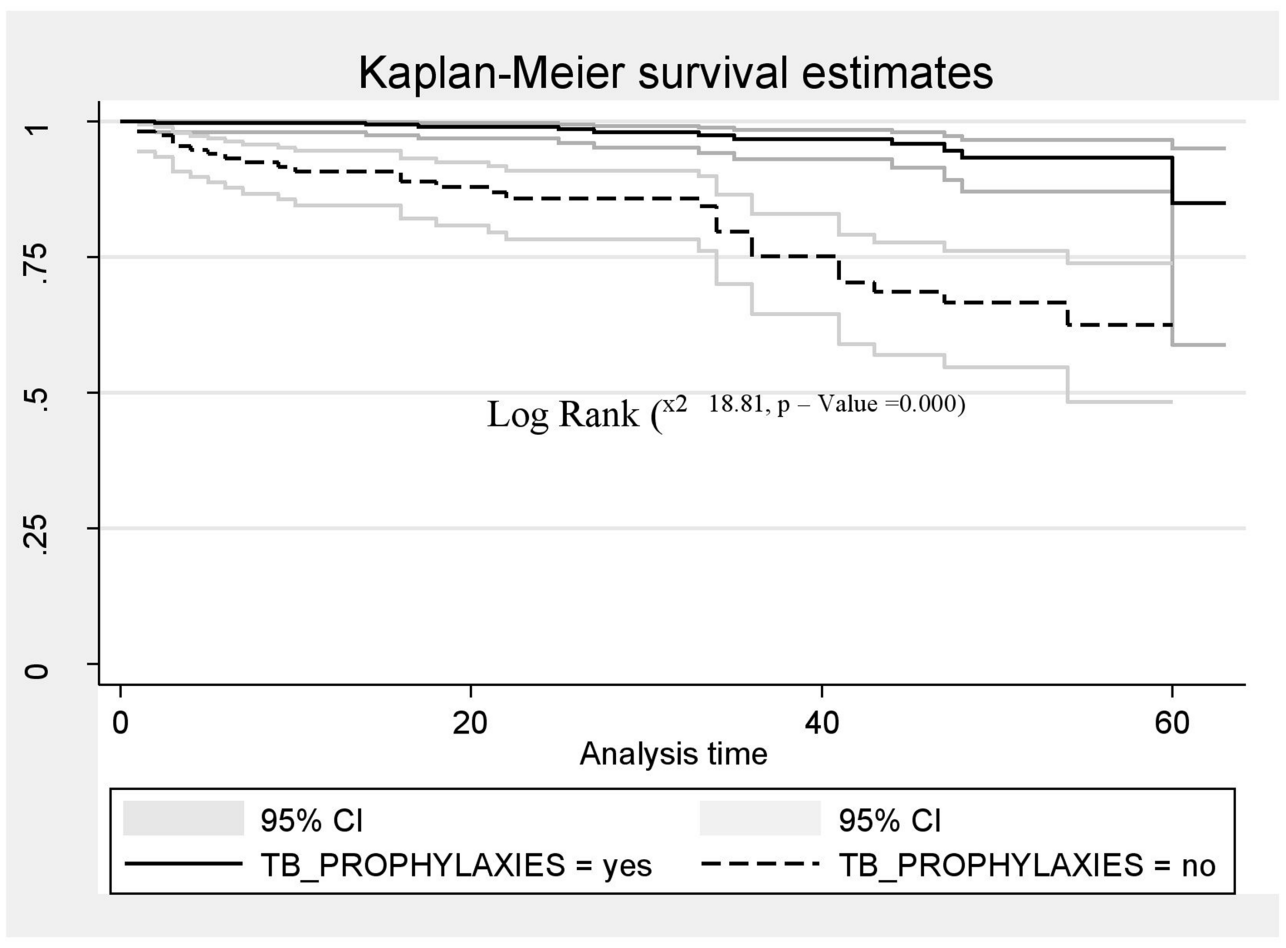
The Kaplan–Meier survival estimates based on TB prophylaxis in the retrospective study of the HIV patients on ART at the public facilities in the HGW Zone, Western Ethiopia, 2024.

**Figure 7:**
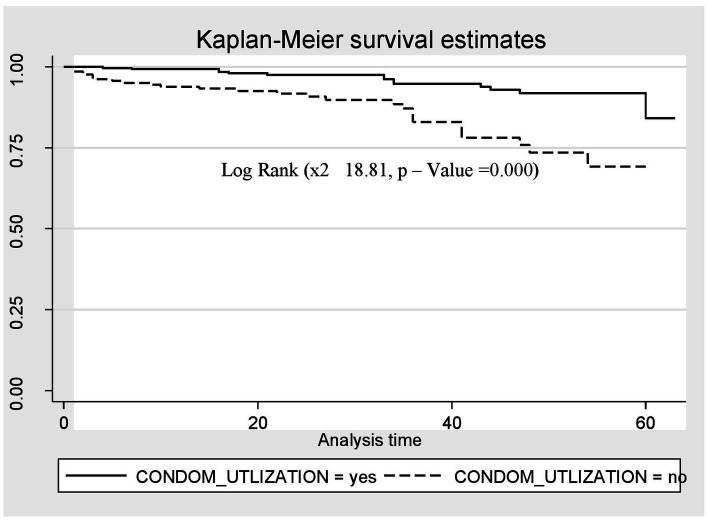
The Kaplan–Meier survival estimates based on non-utilization of condoms in the retrospective study of the HIV patients on ART at the public facilities in HGW, Western Ethiopia, 2024.

**Figure 8:**
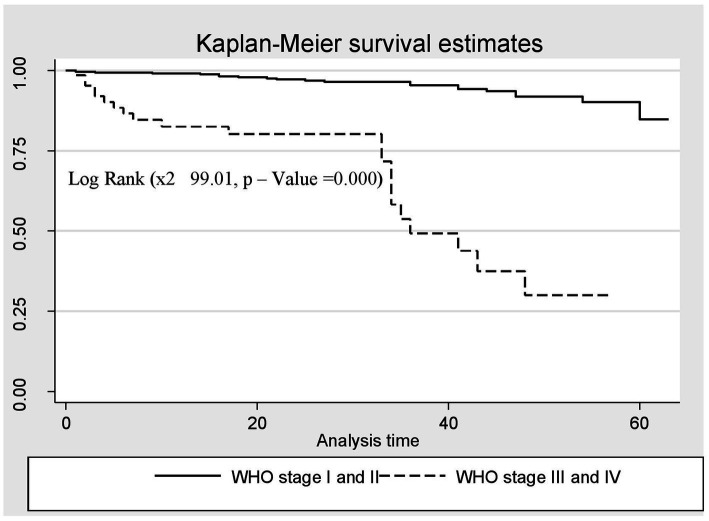
The Kaplan–Meier survival estimates based on WHO clinical staging status in the retrospective study of the HIV patients on ART at the public facilities in HGW, Western Ethiopia, 2024.

A Kaplan–Meier estimation technique was used to estimate survival time. The overall Kaplan–Meier survival curve depicted a gradual decline over the first 30 months, indicating that most deaths occurred slowly during this period. HIV-related diarrhea, failure to adhere to TB prophylaxis, non-utilization of condoms, and WHO staging status were variables that showed significance in the log-rank test (Figures 5–8).

### Predictors of death in the HIV-infected patients (mortality)

To determine the independent predictors of time to death among the patients on ART, a bivariate Cox proportional hazards model was utilized. Using an independent predictor variable coded as 1 for success or death and as 0 for censored, a bivariate Cox proportional hazards model was applied to identify independent predictors of death. Independent variables with *p*-values less than 0.25—such as age, sex, marital status, HIV-related diarrhea, herpes zoster, mycosis, pneumonia, TB prophylaxis, condom utilization, alcohol consumption, substance use, adherence to ART, functional status, and WHO staging status—were considered candidate variables for the multivariable Cox proportional hazard analysis to identify significant predictors associated with time to death among the patients on ART. In the final multivariable Cox proportional hazards regression analysis, HIV-related diarrhea, TB prophylaxis, condom utilization, and WHO staging status were identified as predictors of death at the 5% significance level. Therefore, the risk of death among the HIV-positive patients who had a history of HIV-related diarrhea was 4.5 times higher (AHR = 4.54;95% CI 1.85–11.13) compared to those who had no history of diarrhea. Similarly, the risk of death among HIV-positive patients who were not supplied with TB prophylaxis was two times higher than those who were supplied with TB prophylaxis (AHR = 2.25; CI: 5.61, 14.03). The risk of death among the HIV-positive patients who were not utilizing condoms was time times higher compared to those who were utilizing condoms (AHR = 2.62; CI: 1.13, 6.08). Furthermore, the risk of death among the HIV-positive patients in WHO stages III and IV was seven times higher compared to those in WHO stages I and II (AHR = 7.02; CI: 3.11, 11.84)(Table 6).

**Table 6:** Bivariate and multivariable Cox regression analysis of predictors of death among HIV-infected Patients receiving ART in Public facilities of HGW zone, Western Ethiopia, 2024.

Variable	Category	Outcome variable		CHR (95% CI)	AHR (95%CI)	*p*-value
		Censored (*n* = 496)	Died (*n* = 42)			
Age	≤ 27 years	133	5	1	1	
	28–35 years	164	21	2.25(0 0.85, 5.99)	1.22(0.39,3.84)	0.739
	36–42 years	108	4	0.63(0.17, 2.37)	43.07(0.07,2.52)	0.350
	≥ 43 years	91	12	2.41(0.85, 6.85)	2.91(0.81,10.46)	0.102
Sex	Male	195	21	1	1	
	Female	301	21	0.68(0.37, 1.24)	1.65(0.68, 4.03)	0.268
Marital status	Never married	64	4	1	1	
	Married	354	28	1.16(0.41,3.37)	1.28(0.39,4.23)	0.682
	Separated	71	3	3.72(0.83,16.76)	3.14(0.47,20.76)	0.064
	Divorced	71	7	2.31(0.65,8.24)	4.17(0 0.92,18.83)	0.364
HIV related Diarrhea	Yes	19	11	6.48(3.24, 12.93)	4.54(1.85,11.13)	0.001***
	No	477	31	1	1	
Herpes zoster	Yes	3	1	3.61(0.49, 26.32)	2.53(13.45,47.65)	0.535
	No	493	41	1	1	
Mycosis	Yes	3	1	3.64(0.87,15.11)	2.08(0.38,11.50)	0.402
	No	493	41	1	1	
Pneumocystis pneumonia (PCP)	Yes	17	6	3.45(1.45, 8.20)	1.89(0 0.68, 5.30)	0.224
	No	479	36	1	1	
TB prophylaxis	Yes	362	11	1	1	0.001***
	No	134	31	7.39(3.71, 14.70)	5.61(2.25,14.03)	
Condom utilization	Yes	308	14	1	1	0.025*
	No	188	28	3.76(1.97, 7.16)	2.62(1.13, 6.08)	
Alcohol Consumption	yes	41	14	1	1	0.931
	No	455	28	4.86(2.53, 9.32)	1.05(0 0.35, 3.16)	
Substance use	Yes	23	8	4.97(2.28, 10.83)	2.54(0.57, 11.32)	0.221
	No	473	34	1	1	
Smoking	Yes			6.95(3.04,15.90)	2.32(0.41,13.06)	0.340
	No			1	1	
ART Adherence	Good	426	27	1	1	
	Fair	20	3	2.19(0 0.67, 7.21)	0.42(0.09 2.034)	0.284
	Poor	50	12	4.14(1.95 8.80)	2.36 (0.94 5.91)	0.066
Functioning status	Working	457	31	1	1	
	Ambulatory	36	8	5.27(2.42, 11.49)	1.83(0.66 5.09)	0.246
	Bedridden	3	3	4.60(1.09, 19.27)	2.72(0 0.61 12.16)	0.191
WHO staging	Stage I and II	451	21	1	1	0.000***
	Stage III and IV	45	21	12.10(6.49, 22.56)	7.04(3.56, 13.91)	

## Discussion

This study revealed that the overall incidence rate of death was 2.81 deaths per 1,000 person-months (95%CI: 2.08, 3.81), which is similar to a study conducted in northwest Ethiopia, which reported an incidence rate of 2.87 per 1,000 child-months (95%CI: 1.94–4.25) (1). This finding is lower compared to a study conducted in Mozambique, with an incidence death rate of 6.86%; a study conducted in rural Kenya, with an incidence rate of12.78 per 100 person-years (95% CI 8.49, 19.23); a model approach study in Ethiopia, with 11 per 1,000 person-months; a study conducted in Addis Ababa of Ethiopia, with an incidence rate of 4.98 per 1,000 child-months (95% CI: 3.67–6.77); and a study conducted in Harari Ethiopia, with an incidence rate of 4.75 per 100 person-years (16, 27–30). This variation might be due to differences in the quality of care, sample size, study setting, and study period. However, this finding is higher than that of a study conducted in South Gondar, Ethiopia, which reported a mortality rate of 2.59 per 1,000 person-years (95% CI = 0.02136–0.031736), as well as a study in Amhara, Ethiopia, which reported a mortality rate of 1.52 per 100 person-years, and a study in Bahirdar, Ethiopia, which reported an incidence density rate of 0.9 per 1,000 child-months (95%CI: 0.6–1.3), (11, 21). This variation might be due to differences in sample size, study area, and the behavior of study participants.

HIV-related diarrhea, failure to adhere to TB prophylaxis, non-utilization of condoms, and WHO staging status were found to be predictors of death among the HIV-positive patients. Therefore, the risk of death among the HIV-positive patients who had a history of HIV-related diarrhea was 4.5 times higher (AHR = 4.54;95% CI 1.85–11.13) compared to those who had no history of diarrhea. This finding is in line with those of studies conducted in Mozambique (27), Serra Leone (31), and Ethiopia (32), which could be because HIV-infected patients in these regions are at high risk of developing diarrheal disease and may be more vulnerable to enteric pathogens. These factors may increase the risk of dying from HIV-related diarrheal diseases. However, this result differs from those of studies conducted in Addis Ababa, Ethiopia, and different parts of Ethiopia (30, 33, 34). This difference might be due to variations in the study area, study setting, sample size, and quality of care.

Similarly, the risk of death among HIV-positive patients who were not supplied with TB prophylaxis was two times higher compared to those who were supplied with TB prophylaxis (AHR = 2.25; CI: 5.61, 14.03). This finding is supported by studies conducted in China, Buno Bedele, and the Amhara region of Ethiopia (20, 31, 35), which may be because TPT provision can help reduce the risk of developing active TB. As the risk of developing TB is higher among those with a compromised immune system, including people living with HIV (PLHIV), lack of TB prophylaxis may increase the likelihood of progressing directly to advanced WHO stages III and IV, or even result in premature death for PLHIV. Overall, the African region accounted for 82% of TB cases among HIV-positive individuals, with some countries in sub-Saharan Africa reporting up to 1.68 million deaths due to TB infection, of which 0.38 million deaths were HIV-positive individuals (36). The provision of isoniazid preventive therapy (IPT) is one of the key public health interventions for the prevention of TB in HIV-infected individuals.

Similarly, the risk of death among the HIV-positive patients who were not utilizing condoms was three times higher compared to those who were utilizing condoms (AHR = 2.62; CI: 1.13, 6.08). This finding is supported by those of studies conducted in China, Ethiopia, and Tanzania (31, 32, 37). Condom utilization was found to be low among people living with HIV/AIDS attending ART clinics in Ethiopia (38). If HIV-positive individuals have sex without a condom, those with a low CD4 count or a higher viral load at the initiation of ART are at risk of transmitting the virus to a seronegative sexual partner or may be at risk of acquiring drug-resistant viral strains themselves. Therefore, the promotion, demonstration, and progressive utilization of condoms, along with awareness creation, are important at all levels.

Furthermore, the risk of death among the HIV-positive patients in WHO stages III and IV was seven times higher compared to those in WHO stages I and II (AHR = 7.02; CI: 3.11, 11.84). This finding is consistent with those of studies conducted in southwestern China and the Amhara region of Ethiopia (39, 40). This consistency could be because the advanced clinical stage indicates significantly weakened immunity, which results in opportunistic infections, a major cause of mortality among HIV-infected patients. However, this finding is not in line with those of studies conducted in Mozambique, Tanzania, China, and some parts of Ethiopia (27, 37, 39, 40). This variation may be due to patient misbehaviors, poor adherence, and the quality of care services. The majority of people living with HIV included in the previous studies were not using condoms, were smokers, consumed alcohol, and had irregular ART drug collection schedules, which may have contributed to their progression to an advanced clinical stage.

### Limitations of the study

The study has some limitations as it relied on secondary data. A significant number of patients dropped out, which might have impacted the study result. Therefore, an investigation into the reason behind the dropout from ART would be highly valuable. In addition, another drawback of a retrospective study using patient registration cards is the potential for bias arising from incomplete data, as participants with missing outcome data may differ significantly from those with complete data. Some important variables were missed despite our efforts to include comprehensive predictors. We welcome further research in this area.

## Conclusion and recommendations

This study revealed that the incidence of death among HIV patients in this study area was higher compared to the national HIV-related death report. Therefore, a history of HIV-related diarrhea, failure to adhere to TB prophylaxis, non-utilization of condoms, and HIV clinical stages III and IV were found to be predictors of time to death. Moreover, this study provides several insights into the fundamental causes of why a patient with HIV/AIDS on ART dies while still on follow-up and therapy.

Patients should strictly adhere to their regular ART regimen and promptly visit a health facility, especially for the treatment of diarrhea and other manifestations of WHO clinical stages. In addition, health facilities should ensure accurate data recording and reporting to address data discrepancies and irregular monitoring. This finding will help minimize the occurrence of opportunistic infections and ensure that patients are ready to receive immediate treatment.

Furthermore, healthcare providers should administer TB prophylaxis and provide condoms to prevent TB co-infection and sexually transmitted infections. Similarly, the Ministry of Health, health sector managers, and supporting partners should focus on supplying preventive therapies for opportunistic infections, such as TB prophylaxes and condom distribution, with a strong emphasis on training and capacity building at both facility and community levels. Finally, researchers should use this study as a baseline and focus on conducting prospective, community-based qualitative research to strengthen the understanding of causality.

## Data Availability

The original contributions presented in the study are included in the article/supplementary material, further inquiries can be directed to the corresponding author/s. The raw data supporting the conclusions of this article will be made available by the authors, without undue reservation.
